# Identifying EEG biomarkers of sense of embodiment in virtual reality: insights from spatio-spectral features

**DOI:** 10.3389/fnrgo.2025.1572851

**Published:** 2025-05-12

**Authors:** Daniela Esteves, Madalena Valente, Shay Englander Bendor, Alexandre Andrade, Athanasios Vourvopoulos

**Affiliations:** ^1^Institute for Systems and Robotics (ISR-Lisboa), Bioengineering Department, Instituto Superior Técnico, Lisbon, Portugal; ^2^Instituto de Biofísica e Engenharia Biomédica, Faculdade de Ciências da Universidade de Lisboa, Lisbon, Portugal

**Keywords:** sense of embodiment, virtual reality, electroencephalography, biomarkers, motor imagery, brain-computer interfaces

## Abstract

The Sense of Embodiment (SoE) refers to the subjective experience of perceiving a non-biological body part as one's own. Virtual Reality (VR) provides a powerful platform to manipulate SoE, making it a crucial factor in immersive human-computer interaction. This becomes particularly relevant in Electroencephalography (EEG)-based Brain-Computer Interfaces (BCIs), especially motor imagery (MI)-BCIs, which harness brain activity to enable users to control virtual avatars in a self-paced manner. In such systems, a strong SoE can significantly enhance user engagement, control accuracy, and the overall effectiveness of the interface. However, SoE assessment remains largely subjective, relying on questionnaires, as no definitive EEG biomarkers have been established. Additionally, methodological inconsistencies across studies introduce biases that hinder biomarker identification. This study aimed to identify EEG-based SoE biomarkers by analyzing frequency band changes in a combined dataset of 41 participants under standardized experimental conditions. Participants underwent virtual SoE induction and disruption using multisensory triggers, with a validated questionnaire confirming the illusion. Results revealed a significant increase in Beta and Gamma power over the occipital lobe, suggesting these as potential EEG biomarkers for SoE. The findings underscore the occipital lobe's role in multisensory integration and sensorimotor synchronization, supporting the theoretical framework of SoE. However, no single frequency band or brain region fully explains SoE. Instead, it emerges as a complex, dynamic process evolving across time, frequency, and spatial domains, necessitating a comprehensive approach that considers interactions across multiple neural networks.

## 1 Introduction

Sense of Embodiment (SoE) refers to the subjective experience of perceiving a non-biological body part, such as a virtual avatar or prosthetic limb, as part of one's own body, created by sensations of being within, owning, and controlling it (Kilteni et al., 2012). The concept was first explored by Botvinick and Cohen (1998), who introduced the Rubber Hand Illusion (RHI), an experiment demonstrating that synchronized tactile and visual feedback could transfer the sensation of touch to a fake limb. This work laid the groundwork for embodiment research, which later extended into Virtual Reality (VR). In VR, individuals could replace their physical bodies with virtual avatars, facilitating the SoE through responsive visual feedback (Guy et al., 2023; Choi et al., 2020b; Škola and Liarokapis, 2023). Several studies have replicated the RHI in virtual environments (VEs) (Lenggenhager et al., 2007; Slater et al., 2008; Yuan and Steed, 2010), confirming that immersive VR can evoke similar ownership illusions, particularly with realistic visual representations. Over time, the virtual limb illusion has expanded into full-body ownership illusions, demonstrating that individuals can embody entire virtual avatars (Slater, 2009; Petkova and Ehrsson, 2008; Vagaja et al., 2024; Guy et al., 2023). Therefore, VR is a relevant tool to manipulate and investigate SoE.

The most recent model defines SoE as comprising three interrelated components: the sense of ownership (SoO), sense of agency (SoA), and sense of self-location (SoSL). When these components align, users experience SoE toward the fake body (Kilteni et al., 2012; Vagaja et al., 2024; Guy et al., 2023). SoO involves attributing sensations to one's body parts, integrating bottom-up sensory inputs (e.g., visual, tactile, proprioceptive) and top-down cognitive expectations (e.g., internal body maps) (Kilteni et al., 2012; Segil et al., 2022; Guy et al., 2023). A stronger SoO arises when external objects resemble the real body, requiring anatomical plausibility and spatial alignment. For example, VR enhances this by offering customizable avatars (Guy et al., 2023; Segil et al., 2022). Regarding SoA, it is the subjective experience of controlling one's movements and their effects on the environment. It involves the feeling of agency (implicit, non-reflective sense tied to action initiation) and judgment of agency (higher-level reasoning based on sensory feedback). So, it arises from sensorimotor integration, requiring temporal and spatial congruence between motor commands and sensory feedback (Kilteni et al., 2012; Segil et al., 2022; Guy et al., 2023). VR enhances SoA with precise tracking and minimal latency, ensuring seamless avatar control (Guy et al., 2023). Finally, SoSL involves the perception of being located within one's body. Unlike the sense of presence, which relates to immersion in the virtual world regardless of body perception, SoSL is tied to the perception of one's physical boundaries. It depends on visuospatial perspective (first- vs. third-person views), vestibular signals (balance, orientation), and tactile inputs across personal, peripersonal, and extrapersonal spaces (Kilteni et al., 2012; Guy et al., 2023). Notably, studies show that first-person perspectives in VR enhance SoSL more effectively than third-person views (Guy et al., 2023). Although these three components are distinct and can be experienced separately, they are interconnected and not easily dissociated neurophysiologically. Still, there is limited understanding of how each subcomponent contributes to the overall SoE or how they interact with one another (Kilteni et al., 2012; Guy et al., 2023; Segil et al., 2022).

Thus, SoE in VR relies on three primary sensory triggers: visuomotor (synchronized visual-motor feedback), visuotactile (alignment of touch and visual input), and visuoproprioceptive (perspective-based cues, such as first- or third-person perspective) (Vagaja et al., 2024; Guy et al., 2023). Beyond sensory factors, task demands, emotions, personality, and social context shape SoE (Guy et al., 2023). The Proteus effect shows how users adapt behavior based on their avatar's attributes, task goals, and personal preferences (Yee and Bailenson, 2007). Emotions and personality traits further modulate the strength of SoE, leading to diverse experiences across individuals. SoE also influences social perceptions, with studies showing that embodying avatars of different races or ages can reduce implicit biases (Peck et al., 2013; Banakou et al., 2016). In this way, SoE is personal and specific, with some individuals suffering profound behavioral changes, such as the Proteus effect (Yee and Bailenson, 2007), while others may show little to no modification in their behavior or perception, highlighting the complexity of this sense (Guy et al., 2023).

SoE in VR and Motor Imagery-based Brain-Computer Interfaces (MI-BCIs) are closely related, as both rely on an individual's ability to perceive and interact with a virtual or external representation of their body. MI-BCIs capitalize on the user's mental ability to imagine motor actions, which are then translated into commands for controlling virtual or robotic representations (Alimardani et al., 2016). The effectiveness of MI-BCIs can be enhanced by embodying a virtual avatar which has been shown to improve motor imagery performance (Vourvopoulos and i Badia, 2016; Vourvopoulos et al., 2022; Amini Gougeh and Falk, 2023). Further, Pérez-Marcos et al. (2009) demonstrated that SoE could be induced using MI- BCIs. This study showed that neurofeedback, delivered via virtual hands performing imagined movements in synchrony with the user's intentions, could enhance embodiment. BCIs are powerful tools for translating brain activity into external commands for computer systems like VR or robotic platforms mainly for restoration or communication for patients with neurological disorders (Daly and Huggins, 2015; Wolpaw et al., 2020; Chen et al., 2023). For patients with severe motor impairments, MI-BCIs provide an essential non-invasive rehabilitation strategy that targets the brain directly, strengthening damaged sensorimotor networks, and promoting neural recovery (Daly and Huggins, 2015; Vagaja et al., 2024; Vourvopoulos et al., 2019; Choi et al., 2020b).

A crucial factor in this improvement is considered to play the SoE, which enhances the effectiveness of VR-based MI-BCI systems. Research demonstrates that virtual hand illusions and MI tasks share similar electrophysiological patterns, particularly Event-Related Desynchronization (ERD) in frontoparietal brain areas (Pfurtscheller and Da Silva, 1999). This suggests that SoE strengthens ERD patterns during MI training, leading to better MI-BCI performance (Vourvopoulos et al., 2022). For instance, studies have found that immersive VR environments with virtual hand feedback significantly enhance user performance by increasing immersion, cortical activation, and BCI usability (Choi et al., 2020a). Although some research highlights the limited benefits of non-immersive VR (Song and Kim, 2019), the overall evidence supports the idea that SoE plays a crucial role in improving MI-BCI efficiency. This is achieved through increased neuroplasticity, higher classification accuracy, and greater patient motivation, making VR-based embodiment a valuable tool for motor rehabilitation (Jeong and Kim, 2021).

Nonetheless, incorporating virtual embodiment into MI-BCI neurorehabilitation is a complex challenge, particularly due to the difficulty of assessing SoE during MI-BCI training. Since SoE is primarily a subjective experience, the most widely accepted method for its evaluation relies on questionnaires and self-reports, typically using Likert scales due to their simplicity (Guy et al., 2023; Segil et al., 2022). Still, the lack of standardized questionnaires across studies complicates comparisons. Efforts to create more uniform assessment questionnaires, such as the 16-item version by Peck and Gonzalez-Franco (2021), have improved comparability but new questionnaires continue to emerge, adding to the complexity (Guy et al., 2023). Furthermore, their reliability is criticized due to personal subjectivity, participant interpretation, and scale biases. These factors make questionnaires not the “gold standard” for SoE assessment and prompt research into more objective methods.

To address this, researchers have explored behavioral measures such as proprioceptive drift, pain perception, intentional binding, and sensory attenuation. However, these metrics typically reflect individual SoE components. For instance, proprioceptive drift is associated with SoSL, while sensory attenuation of self-touch and intentional binding are closely linked to SoA. Additionally, contradictory reports on their reliability further introduce uncertainties regarding these behavioral changes as SoE measurement methods (Guy et al., 2023; Segil et al., 2022). Physiological measures have also been investigated, including skin temperature and conductance. Lower skin temperature has been linked to disownership, while higher temperature correlates with SoE due to autonomic responses. Yet, results are inconsistent and influenced by uncontrolled factors like room temperature (Segil et al., 2022). Similarly, skin conductance reflects autonomic responses like sweating. Increased conductance when an artificial hand is threatened suggests SoE, but external factors (e.g., participant variability, repeated exposure) affect reliability, and contradictory findings limit its usability as an objective measure (Segil et al., 2022).

Neurophysiological research using Electroencephalography (EEG) has explored brain activity associated with SoE, primarily focusing on spectral analysis (Segil et al., 2022). Most studies examine changes in frequency bands, such as Theta, Alpha, Beta, and Gamma, with fewer investigating correlations involving Delta activity. Increased Theta power has been linked to embodied situations, such as Theta power in response to observing avatar errors when embodied (Pavone et al., 2016), or greater Theta event-related synchronization (ERS) in left frontocentral areas during high levels of SoA (Pavone et al., 2016). However, Hansford et al. (2023) observed greater Theta activity in the parietal area during incongruent visuotactile stimuli (disembodied situations), suggesting a higher cognitive workload to assimilate incongruent inputs. In contrast, other studies report no significant change in Theta power during SoE (Li et al., 2023) highlighting inconsistencies in the literature. The most consistent EEG finding in the literature is the correlation between increased Alpha ERD, particularly over central and parietal lobes, and a strong SoE (Alchalabi et al., 2019; Raz et al., 2020; Evans and Blanke, 2013; Shibuya et al., 2021; Kang et al., 2015; Sciortino and Kayser, 2022a,b; Faivre et al., 2017; Rao and Kayser, 2017; Della Longa et al., 2021; Shibuya and Ohki, 2023; Shibuya et al., 2018). While these changes are typically observed in somatosensory areas without lateralization (Sciortino and Kayser, 2022b), only three studies reporting these changes were implanted in VR settings (Alchalabi et al., 2019; Raz et al., 2020; Evans and Blanke, 2013). Nonetheless, contradictory results persist, with some studies reporting no changes in Alpha power during SoE (Li et al., 2023), or even a decrease in Alpha during SoO in eyes-closed resting state but no changes during the eyes-open condition (Hsu et al., 2022). In addition, Lenggenhager et al. (2011) suggested that increased Alpha suppression correlates with high levels of SoSL but not necessarily with SoE itself. Regarding Beta power, studies in non-VR settings often report a decrease during SoE, particularly in central, left sensorimotor, bilateral temporal, and occipital regions (Shibuya et al., 2021; Kang et al., 2015; Sciortino and Kayser, 2022b; Rao and Kayser, 2017). Yet, Faivre et al. (2017) observed a correlation between SoE and increased Beta power over frontotemporal areas in no-VR settings. Interestingly, several VR studies have failed to detect significant changes in Beta power during embodiment illusions (Alchalabi et al., 2019; Evans and Blanke, 2013; Li et al., 2023; Hansford et al., 2023), suggesting that the reported Beta changes associated with SoE are related to the non-immersive settings. Lastly, changes in Gamma activity have also been reported, with increased Gamma power in frontal and central regions, especially in the somatosensory cortex and left parietal region, being related with strong SoE (Li et al., 2023; Hiramoto et al., 2017; Hansford et al., 2023). SoE has also been linked to increased Gamma connectivity (Li et al., 2023; Faivre et al., 2017). However, some studies in VR settings found no correlation between SoE and Gamma activity (Alchalabi et al., 2019; Evans and Blanke, 2013). Moreover, lower Gamma power over frontotemporal and central areas has been associated with high SoA (Kang et al., 2015), complicating the understanding of the relationship between SoE, its components, and Gamma activity.

Other changes, such as alterations in somatosensory evoked potentials (SEPs) (Aspell et al., 2012; Sakamoto and Ifuku, 2021), other Event-Related Potentials (ERPs) components (González-Franco et al., 2014; Galigani et al., 2021; Rao and Kayser, 2017), overall power spectral density (PSD) (Blefari et al., 2011), fractal dimension (Veillette et al., 2023), error-related potentials (ErrPs) (Porssut et al., 2023; Raz et al., 2020; Pavone et al., 2016), and altered signals between the brain and muscles (Li et al., 2023) during SoE illusion have been reported. These findings suggest complex brain activity during embodiment illusions, with potential distinctions from non-embodiment conditions.

Despite growing interest in EEG-based biomarkers of the SoE, a significant research gap remains, particularly in VR settings, where neurophysiological correlates of SoE are still largely underexplored. While some studies have identified promising EEG patterns, inconsistencies across findings—driven by methodological variability in paradigms, data collection, and EEG setups—have prevented the establishment of a definitive biomarker. The lack of standardized procedures for inducing and assessing SoE further hampers progress, underscoring the need for universally accepted protocols and objective metrics beyond subjective questionnaires. A reliable EEG biomarker would bridge the gap between self-reports and neurophysiological evidence, offering valuable applications in fields like MI-BCIs for rehabilitation, where virtual embodiment could enhance patient engagement and optimize neurorehabilitation outcomes. Additionally, such a biomarker would be highly beneficial in VR research, enabling more precise assessments of avatar embodiment and improving the design of immersive virtual experiences. Addressing these challenges, this study investigates if there is a reliable EEG-based biomarker that correlates with the SoE in VR.

## 2 Methods

This study integrates both the dataset from Vagaja et al. (2024), extended by newly recorded data with a total of 41 healthy participants. The overall experimental setup and data acquisition methods remained largely consistent, acquired at the same location and equipment.

### 2.1 Participants

The first dataset includes data from 26 participants (16 female, 61.54%; 10 male, 38.46%) with a mean age of 24.12 ± 5.00 years. Participants were randomly assigned to either the control group (5 males, 8 females) or the embodied group (6 males, 7 females) in a between-subject study design. Further details on the experimental setup can be found at the original paper (Vagaja et al., 2024).

The newly recorded dataset comprised data from 15 participants (8 females, 53.33%; 7 males, 46.67%), with an average age of 26.00 ± 6.47 years. All participants were right-handed, as confirmed by the Edinburgh Handedness Inventory (EHI) (Oldfield, 1971), with an average laterality quotient (LQ) of 67.11 ± 24.84. Participants were screened to ensure they had no known neurological conditions and possessed either normal or corrected vision, as the VR headset can accommodate prescription glasses. Before participating, all individuals provided written informed consent following the ethical guidelines of the 1964 Declaration of Helsinki.

### 2.2 Experimental design

**Figure 1:**
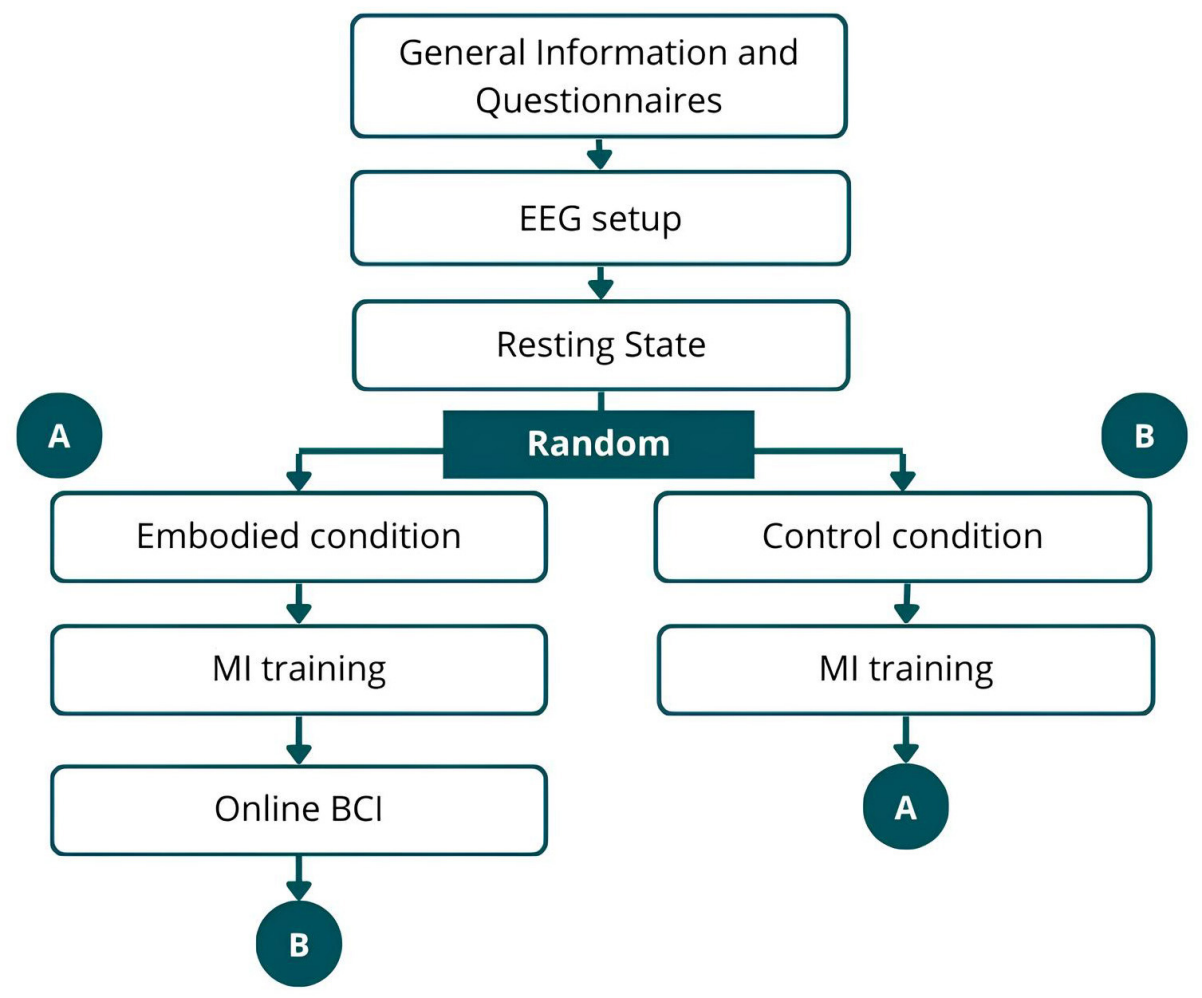
Schematic representation of the data collection procedure. After general information, EEG setup, and resting-state recording, participants were randomly assigned to either embodied condition (path A) or control condition (path B). After completion, they switched to the other path, ensuring all phases were completed.

**Figure 2:**
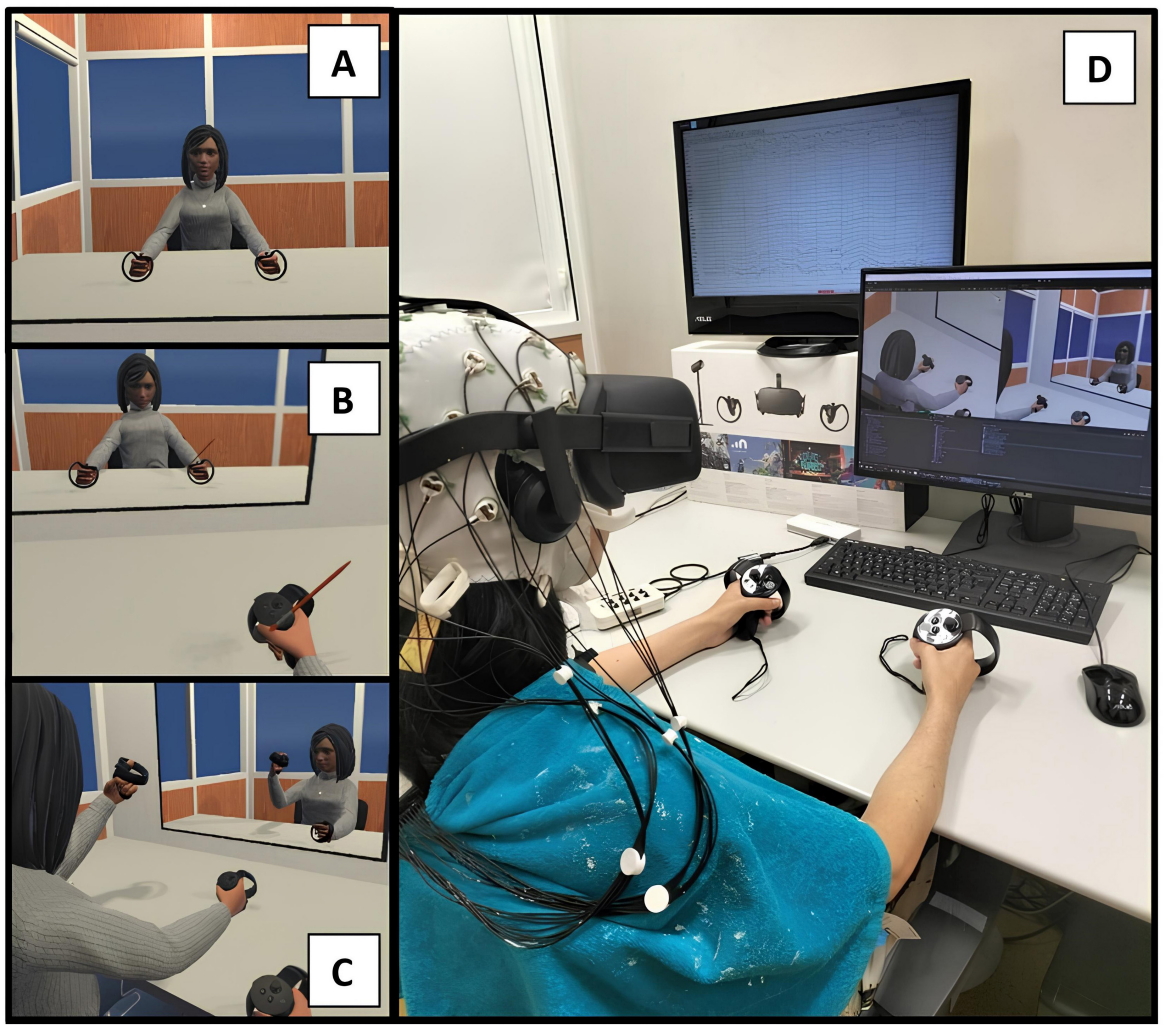
Experimental design with a female virtual avatar. **(A)** Represents the VE during the exploration section of the embodied condition, while **(B)** represents the brushing part (virtual hand illusion) of the same condition. **(C)** Shows the VE during the exploration part of the control condition. **(D)** Illustrates a female participant during the control condition, with the EEG and VR headset set up.

For the newly recorded dataset, a within-subjects design was employed to control for individual variability and mitigate potential confounding factors that are inherent in between-groups design (Vagaja et al., 2024). The protocol involved the participant preparation, EEG setup, and seven recording phases (Figure 1), with the order of embodied and control conditions randomized. The recording phase consisted of resting-state EEG recording, after which the VR headset was carefully placed over the electrodes. Participants were then randomly assigned to either embodied condition or control condition (Figure 1). Following the embodied/control conditions, participants underwent MI training using an offline BCI. After MI training in the embodied condition, they completed the MI training in online BCI. Upon completing one branch, participants proceeded to the other, ensuring that each subject underwent all phases. Figure 2 illustrates the VE as seen by participants during different phases.

To address the goal of this study, specifically, identifying a general EEG-based biomarker of the SoE, this research focused exclusively on data from the Embodied and Control conditions to examine potential differences in EEG activity during the SoE illusion compared to disembodied states. Consequently, data from the MI training and online BCI phases were not included in the analysis. Although these phases were part of the experimental protocol, they fall outside the scope of the present study. Moreover, as previously reported by Vagaja et al. (2024), prior SoE induction is not expected to significantly impact MI-BCI performance.

- **Information and EEG setup:** Relevant information was provided to the participants, who then completed the demographic questionnaire. Additionally, the Vividness of Movement Imagery Questionnaire (VMIQ-2) was administered to assess imagery ability across three perspectives: Internal Visual, External Visual, and Kinesthetic (Roberts et al., 2008). Following this, the EEG setup was performed (Figure 2), using conductive gel to ensure electrode impedance remained below 10 KOhm.
- **Resting-state EEG recording:** This phase involved recording EEG signals for 4 min, divided into 2 min of eyes-open resting state followed by 2 min of eyes-closed resting state.
- **Embodied condition:** Participants entered the VE, viewing a gender-matched avatar from a first-person perspective (visuoproprioceptive trigger). For 3 min, the participants explored the environment, namely looking around, seeing their reflexes in the mirror in front of them, and moving their virtual hands, head, and torso without moving the chair, while seeing the movements of the avatar synchronized with their own (visuomotor trigger). Nonetheless, participants were previously instructed to move slowly and smoothly to minimize motion artifacts. After these 3 min of exploration, the participant was asked to remain still and focus on the right hand. A virtual brush appeared and began stroking the virtual hand for 2 min, synchronized and spatially congruent with the experimenter brushing the participant's real hand (visuotactile trigger). The participant focused on the brush stroking the virtual hand, and minimized movements. After 2 min, the participants exited the VE and answered verbally to an embodiment questionnaire.
- **Control condition:** In this condition, participants entered the VE and viewed a gender-matched avatar from a third-person perspective, disrupting visuoproprioceptive triggers. Like the embodied condition, they explored the environment for 3 min, moving their virtual hands, head, and torso with slow, smooth movements, without dislocating the chair. However, the avatar's movements were independent of the participant's real movements, creating incongruent visuomotor triggers. Then, participants were asked to focus on their right hand, minimizing movements. Unlike the embodied condition, no virtual brush appeared. Instead, participants only felt their real hand being brushed, creating incongruent visuotactile triggers. After 2 min, the brushing stopped, participants exited the VE, and then responded verbally to the embodiment questionnaire.
- **MI training:** Participants entered a VE similar to the one used in the embodied and control phases but without the virtual mirror, enabling full focus on the virtual hands. They had to focus on a cross between two virtual hands, and when an arrow appeared pointing to one hand, they were to imagine grasping the indicated hand (MI task) without actual physical movement. The MI training consisted of 30 randomly presented trials, with 15 trials per class (left/right-hand grasp). Each trial consisted of a 5-second rest period followed by a 5-second MI task period. When the arrow appeared (visual cue), participants repeatedly imagined grasping the indicated hand while observing the corresponding virtual hand performing the movement.
- **Online BCI:** Participants re-entered the VE used in the MI training phase and repeated the MI training. However, during this phase, feedback was provided in real-time by a machine learning classifier trained on data collected during the MI training phase posterior to the embodied condition phase.

### 2.3 Experimental setup

#### 2.3.1 EEG equipment and acquisition

Participants were prepared with 32 active electrodes arranged according to the international 10–20 system, with the the reference electrode placed on the left mastoid. EEG signals were recorded at a sampling rate of 250 Hz using a wireless EEG amplifier (LiveAmp, Brain Products GmbH, Gilching, Germany). Signal acquisition was managed via BrainVision Recorder software (Brain Products GmbH, Gilching, Germany).

#### 2.3.2 VR scene and equipment

The experiment was conducted in the same VE used in the first dataset (Vagaja et al., 2024), and it is freely available online.^1^ Participants performed the tasks while seated at a virtual desk, facing a mirror during the SoE induction/disruption phases, within a virtual room designed to replicate their real-world surroundings. They interacted with the VE through a gender-matched avatar. The VE was developed using the Unity 3D game engine, with avatars created via Ready Player Me2. Participants utilized an Oculus Rift CV1 headset, manufactured by Oculus VR (a subsidiary of Meta, Inc., United States), equipped with Oculus Touch controllers and Constellation sensors to interact with the VE. A video from the first study is available online.^2^

### 2.4 Embodiment and presence questionnaires

Both isolated datasets followed the guidelines of Peck and Gonzalez-Franco (2021) for a questionnaire to evaluate the SoE illusion during the Control and Embodied phases, which consists of 16 questions (E1-E16). Six features were derived by averaging specific items, capturing the three main components of SoE (SoO, SoA, and SoSL). Although SoSL is not explicitly calculated as a separate metric, it is inherently incorporated into these features. Specifically, SoSL is indirectly assessed through features such as the virtual body's resemblance to the real body (Appearance), its spatial positioning, responsiveness to movements (Response), and the integration of sensory inputs (Multi-Sensory Integration). Additionally, five questions (P1–P5) adapted from the Multimodal Presence Scale (MPS) by Makransky et al. (2017) were used to assess the sense of presence (Table 1). This allowed the evaluation of both senses according to recent guidelines, using a 7-point Likert scale. In total, seven variables were computed from this questionnaire by averaging specific items.

- Appearance = (E1 + E2 + E3 + E4 + E5 + E6 + E9 + E16)/8
- Response = (E4 + E6 + E7 + E8 + E9 + E15)/6
- Ownership = (E5 + E10 + E11 + E12 + E13 + E14)/6
- Multi-sensory = (E3 + E12 + E13 + E14 + E15 + E16)/6
- Agency = (E3 + E13)/2
- Embodiment = (Appearance + Response + Ownership + Multi-sensory)/4
- Physical presence = (P1 + P2 + P3 + P4 + P5)/5

**Table 1:** Embodiment questionnaire utilized during data acquisition to access SoE illusion induction/break and sense of physical presence during the Control and Embodied phases.

**ID**	**Questions**
E1	I felt out of my body.
E2	I felt as if my real body were drifting toward the virtual body or as if the virtual body were drifting toward my real body.
E3	I felt as if the movements of the virtual body were influencing my own movements.
E4	It felt as if my real body were turning into the virtual body.
E5	At some point it felt as if my real body was starting to take on the posture or shape of the virtual body that I saw.
E6	I felt like I was wearing different clothes from when I came to the laboratory.
E7	I felt like the form or appearance of my real body had changed.
E8	I felt a realistic sensation in my hand when I saw the brush touching the virtual hand.
E9	I felt that my own body could have been affected by the virtual world.
E10	I felt as if the virtual body was my body.
E11	At some point it felt that the virtual body resembled my own (real) body in terms of shape, skin tone or other visual features.
E12	I felt as if my body was located where I saw the virtual body.
E13	I felt like I could control the virtual body as if it was my own body.
E14	It seemed as if I felt the touch of the brush in the location where I saw the virtual hand touched.
E15	It seemed as if the touch I felt was caused by the brush touching the virtual hand.
E16	It seemed as if my hands were touching the virtual desk.
P1	The virtual environment seemed real to me.
P2	I had a sense of acting in the virtual environment, rather than operating something from outside.
P3	My experience in the virtual environment seemed consistent with my experiences in the real world.
P4	While I was in the virtual environment, I had a sense of “being there.”
P5	I was completely captivated by the virtual world.

The questionnaire was answered following a 7-point Likert scale. This questionnaire was adapted from Peck and Gonzalez-Franco (2021) and Makransky et al. (2017).

### 2.5 Data analysis

This study analyzed the Embodied (where SoE was induced) and Control (where SoE was disrupted) conditions. All EEG signals were processed using the EEGLAB toolbox (v2023.1) in MATLAB R2022a.

#### 2.5.1 EEG signal pre-processing

The EEG signals were initially downsampled to 125 Hz using EEGLAB's *pop*_*r*_*esample*.*m* function, which automatically applies anti-aliasing. This was followed by bandpass filtering between 1 and 40 Hz. Next, Artifact Subspace Reconstruction (ASR) was applied to clean the signal (Chang et al., 2018), removing channels with prolonged flatening (over 5 seconds), artifacts in more than 15% of data windows, low correlations (< 0.5) with other channels, or excessive line noise. A burst criterion of 10 standard deviations was used to detect and address artifacts without applying high-pass filtering or segment removal. Channels removed during the process were interpolated, and the data were then re-referenced to the common average. Independent Component Analysis (ICA) was performed, using ICLabel to identify components with more than 90% probability to be eye or muscle artifacts for rejection (Pion-Tonachini et al., 2019). Moreover, all components were manually inspected to ensure the removal of remaining artifact components. Lastly, the first 110 seconds during the brush stroking (virtual hand illusion/disruption) were extracted for further analysis.

#### 2.5.2 Frequency analysis

The power spectrum was calculated for each electrode during the Control and Embodied conditions across all participants and then divided into Delta (approximately 0.3–4 Hz), Theta (4–8 Hz), Alpha (8–13 Hz), Beta (13–30 Hz), and Gamma (above 30 Hz) bands. The average absolute power within each band was computed, and normalized as a percentage of the total power within each electrode. Subsequently, the scalp was divided into anatomical lobes, including Frontal (Fp1, Fp2, F7, F8, F3, F4, and Fz), Central (FC5, FC6, FC1, FC2, C3, C4, Cz, CP5, CP6, CP1, and CP2), Temporal (FT9, FT10, T7, T8, TP9, and TP10), Parietal (P7, P8, P3, P4, and Pz), and Occipital (O1, O2, and Oz). Next, the median power of all electrodes within each lobe was calculated for each frequency band.

#### 2.5.3 Linear modeling

Linear models were developed to analyze the relationship between embodiment strength (measured by embodiment scores resulting from the questionnaire) and frequency band power. Two approaches were applied: Linear Regression (LR) and Linear Mixed Effects (LME) models (Pinheiro and Bates, 2000). The LR model served as a simple baseline but does not account for intra-group variability, being less robust when population subgroups are a factor. To address this, the LME model included both fixed and random effects, capturing individual variations within subgroups. Additionally, while LR used simple linear equations (Equation 1), LME explored different variable interactions and random factors to identify the best-fitting equation. Initially, the linear models did not differentiate between frequency bands; however, given the distinct ranges of power across bands and the possible band-specific responses to SoE illusion, separate models for each band were also considered for a more precise analysis.


(1) $$\begin{array}{l} BandPower=EmbodimentScore*\beta_{1}+\beta_{0} \end{array}$$

#### 2.5.4 Statistical analysis

To select the appropriate statistical methods for comparing conditions (Control vs. Embodied), normality and homoscedasticity of the bands' power were evaluated using the Kolmogorov-Smirnov and Levene tests, respectively. Nonetheless, while some features satisfied normality and homoscedasticity criteria, results were inconsistent between features. Thus, non-parametric tests were used to ensure methodological consistency. All condition comparisons were conducted in MATLAB using the Mann-Whitney U test with a significance level of 0.05. For linear models, the best LME equations were selected based on the lowest Akaike Information Criterion (AIC) (Akaike, 1974), with the Bayesian Information Criterion (BIC) (Schwarz, 1978) also considered for additional validation. Once the final models were selected, the quality of the model was assessed using adjusted-R^2^ (Chicco et al., 2021). The fitted models also provided *p*-values for each predictor, directly indicating their statistical relevance in predicting the response variable.

#### 2.5.5 Machine learning models

Binary classification machine learning models were developed to assess whether it is possible to identify SoE situations based on specific frequency features. For this purpose, the previous pre-processed EEG signals were used, and multiple datasets were prepared to test the models' performance.

The pre-processed signals were truncated to the 0 to 90-second range and then segmented into windows of varying lengths: 2, 3, 5, 6, 9, 10, 15, 18, 30, 45, and 90 seconds. This means that the complete signal for each trial was divided into consecutive, non-overlapping time windows of a fixed duration. The analysis used the first 90 seconds of each trial to allow for a wider range of window lengths, all being divisors of the total segment duration, thereby ensuring consistent use of the entire available data. Within each window, the power spectrum for each electrode was calculated and divided into the frequency bands (Delta, Theta, Alpha, Beta, and Gamma). The power of each band was then normalized as a percentage of the total power within the respective electrode, following the methodology used in the earlier frequency analysis. Consequently, 11 datasets were created (with the different time windows segmentation), each comprising 32 *electrodes* × 5 *frequency bands* × *number of signal segments* features. This approach allowed for a detailed exploration of the time effect on the illusion, as analyzing datasets of different window lengths could reveal whether time variations hold useful information about SoE and its dynamics over time. Furthermore, six machine learning models were employed to identify the best-performing approach, namely, Decision Tree (DT), Random Forest (RF), Naive Bayes (NB), k-Nearest Neighbors (kNN), Support Vector Machine (SVM), and Multi-Layer Perceptron (MLP).

The same fitting process was applied for all datasets and model types. First, the datasets were analyzed to identify the presence of outliers. The samples considered outliers in multiple features were manually selected for removal. Next, each dataset was divided into 80% for training and 20% for testing in a stratified division to ensure both follow the same class distribution (50%/50%). For kNN, SVM, and MLP models, the following step consisted of Z-score data normalization, since these models handle data within similar ranges better. The remaining models used the data without normalization. Afterward, hyperparameter optimization was conducted on the training set using Bayesian optimization combined with 5-fold cross-validation. The parameters tested for each model are summarized in Table 2. After selecting the optimal parameters, the final models were trained on the entire training set and evaluated on the test set. The accuracy metric was used for the models' performance evaluation.

**Table 2:** Representation of all parameters and respective ranges tested in the different machine learning models trained during hyperparameter optimization.

**Model**	**Paramters**
DT	“MaxNumSplits”: [5, 55] “MinLeafSize”: [2, 6] “SplitCriterion”: {“gdi,” “deviance”}
RF	“NumTrees”: [50, 200] “MaxNumSplits”: [5, 55] “MinLeafSize”: [2, 6]
NB	“Distribution”: {“normal,” “kernel”} “Kernel”: {“normal,” “box,” “epanechnikov,” “triangle”}
kNN	“NumNeighbors”: [2, 20] “Distance”: {“euclidean,” “seuclidean,” “cityblock,” “chebychev,” “hamming,” “cosine,” “minkowski,” “correlation”} “DistanceWeight”: {“equal,” “inverse,” “squaredinverse”}
SVM	“Kernel”: {“linear,” “gaussian,” “polynomial”}, “BoxConstraint”: [0.01, 50] “PolynomialOrder”: [2, 6] “KernelScale”: [0.01, 100]
MLP	“NHiddenLayers”: [1, 5] “NNeuronsSize”: [80, 240] “TrainFcn”: {“trainrp,” “traincgf,” “trainscg,” “traincgb”}

Post-classification, the best-performing model and dataset combination were selected for feature importance analysis. This analysis aimed to identify the most relevant features for classifying SoE, potentially uncovering biomarkers. Feature selection methods specific to the chosen model were applied to determine these critical features.

## 3 Results

### 3.1 Vividness, sense of embodiment and presence

In both studies, the SoE illusion was successfully elicited. Specifically, in the Vagaja et al. (2024) dataset, participants reported a significantly stronger embodiment in the Embodied condition compared to the Control condition (Control: 3.978; Embodied: 5.139; *p*-value = 0.008). A similar pattern was observed when observing only the newly recorded dataset (Control: 3.253; Embodied: 5.081; *p*-value = 0.001), confirming the effectiveness of the embodiment manipulation in both cases. Given this consistency, combining the datasets into a single, larger dataset (combined dataset) is feasible to increase statistical power.

**Figure 3:**
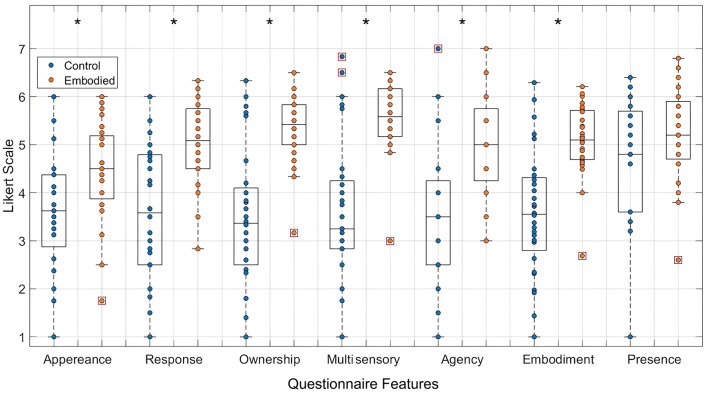
Distribution of features extracted from the embodiment questionnaire, specifically appearance, response, ownership, multi-sensory integration, agency, embodiment, and presence, in a 7-point Likert Scale. Values for the Control condition are represented in blue, while the Embodied condition is in orange. Features with statistically significant differences between conditions, as determined by the Mann-Whitney U test, are marked with an asterisk (*) (*p*-value < 0.05). Each box plot shows the median, interquartile range, and outliers (denoted by red boxes).

The combined dataset demonstrates significant differences in appearance (Control: 3.674; Embodied: 4.460; *p*-value = 0.007), response (Control: 3.557; Embodied: 5.018; *p*-value = 0.001), ownership (Control: 3.485; Embodied: 5.357; *p*-value = 0.000), multi-sensory integration (Control: 3.643; Embodied: 5.595; *p*-value = 0.000), agency (Control: 3.571; Embodied: 5.054; *p*-value = 0.001), and embodiment scores (Control: 3.590; Embodied: 5.108; *p*-value = 0.001), with higher scores in the Embodied condition (Figure 3). These results indicate that the Embodied condition induced a stronger SoE, while the Control condition effectively disrupted this sense. Moreover, both conditions also induced a sense of presence in the virtual environment though presence scores were slightly higher in the Embodied condition (Control: 4.636; Embodied: 5.179; *p*-value = 0.121).

### 3.2 Changes in EEG band power during embodiment illusion

**Figure 4:**
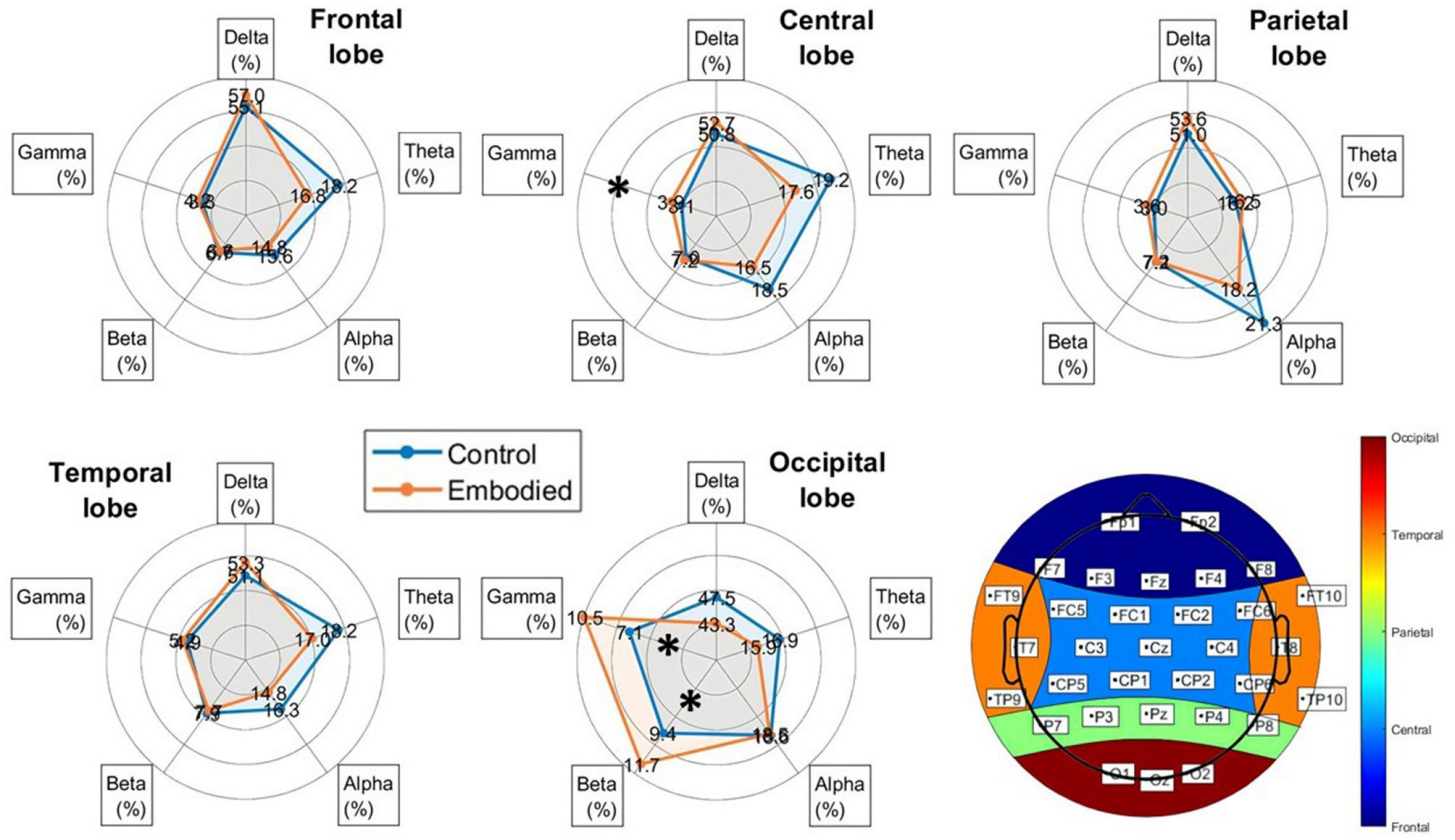
Radar plots of power distribution across EEG frequency bands (Delta, Theta, Alpha, Beta, and Gamma) for each brain lobe, added by the representation of the scalp's lobe division (bottom-left corner), showing the electrodes corresponding to each lobe. Power is expressed as the median percentage relative to the total power within each lobe. The blue line represents the Control condition, while the orange line represents the Embodied condition. Bands with statistically significant differences between groups, as determined by the Mann-Whitney U test (*p* < 0.05), are marked with an asterisk (“*”).

Frequency analysis revealed a slight increase in Delta power during the embodied illusion, except for a non-significant decrease in the occipital lobe (Figure 4). The Theta band exhibited non-significant decreases across the frontal, central, temporal, and occipital lobes during the Embodied condition. Similarly, Alpha power showed non-significant reductions over the frontal, central, parietal, and temporal regions (Figure 4; Table 3). The most notable results were observed in the Beta and Gamma bands. In the Beta band, a significant power increase was found in the occipital lobe during the Embodied condition compared to the Control condition (Control: 9.378%; Embodied: 11.699%; *p* = 0.045; Table 3), while it remained largely unchanged across the rest of the scalp. Lastly, the Gamma band showed a significant power increase in both the central (Control: 3.115%; Embodied: 3.916%; *p* = 0.045; Table 3) and occipital (Control: 7.061%; Embodied: 10.469%; *p* = 0.037; Table 3) lobes during the Embodied condition. An increase in Gamma power was also observed in the parietal lobe, though this change did not reach statistical significance (Figure 4). Overall, the illusion was characterized by increased Delta power (except for a decrease in the occipital lobe), decreased Theta and Alpha power across most of the scalp, increased Beta power in the occipital lobe, and increased Gamma power in the centro-parietal and occipital regions.

**Table 3:** Power of each EEG band (Delta, Theta, Alpha, Beta, and Gamma) as a percentage of total power within each brain lobe, along with Mann-Whitney U-test results (U-statistics and *p*-values) comparing Control and Embodied conditions.

**Lobe**	**Band**	**Control (%)**	**Embodied (%)**	**U-stats (p-value)**
**Frontal**	**Delta**	55.119	57.008	764 (0.583)
	**Theta**	18.232	16.816	914 (0.058)
	**Alpha**	15.565	14.770	796 (0.980)
	**Beta**	6.689	6.554	765 (0.594)
	**Gamma**	3.849	4.154	715 (0.176)
**Central**	**Delta**	50.800	52.689	747 (0.408)
	**Theta**	19.208	17.610	899 (0.100)
	**Alpha**	18.545	16.525	857 (0.338)
	**Beta**	7.006	7.218	746 (0.399)
	**Gamma**	3.115	3.916	675 (0.045*)
**Parietal**	**Delta**	50.967	53.563	739 (0.338)
	**Theta**	16.171	16.451	778 (0.749)
	**Alpha**	21.303	18.214	855 (0.355)
	**Beta**	7.153	7.127	780 (0.774)
	**Gamma**	3.017	3.562	694 (0.090)
**Temporal**	**Delta**	51.139	53.337	734 (0.298)
	**Theta**	18.228	17.044	871 (0.235)
	**Alpha**	16.345	14.839	848 (0.517)
	**Beta**	7.897	7.705	821 (0.712)
	**Gamma**	4.922	5.231	743 (0.372)
**Occipital**	**Delta**	47.548	43.294	864 (0.283)
	**Theta**	16.867	15.869	862 (0.298)
	**Alpha**	18.646	18.509	782 (0.799)
	**Beta**	9.378	11.699	675 (0.045*)
	**Gamma**	7.061	10.469	670 (0.037*)

Significant differences are marked by an asterisk (“*”).

### 3.3 Relationship between embodiment scores and power across frequency bands

The fitted LR and LME models aimed to examine the relationship between embodiment strength and EEG power changes across frequency bands. The initial analysis evaluated correlations using overall frequency power. Among the models tested, the LME model (Equation 2) yielded the lowest AIC (8757.109) and BIC (8903.947), outperforming the LR model (Equation 1), which exhibited higher AIC (12010.492) and BIC (12020.980) values. The LR model demonstrated poor fit (adjusted R^2^ = –0.001), whereas the LME model showed relatively good fit (adjusted R^2^ = 0.904). Despite this, neither model revealed a significant relationship between embodiment scores and general EEG power (LME: *p*-value = 0.872; LR: *p*-value = 0.961; Table 4).


(2) $$\begin{array}{l} Power=Embodiment+Band*Lobe+\left(1|Subject\right) \end{array}$$

**Table 4:** Results of LME and LR statistical models evaluating the relationship between embodiment score, and EEG frequency bands' power.

**Outcome (Model)**	**Models variables**				**Model evaluation**		
	**Variable**	**Estimate**	**t-Statistics**	***p*** **-value**	**AIC**	**BIC**	**Adjusted-R** ^2^
Bands (LME)	Embodiment	–0.018	–0.161	0.872	8,757.109	8,903.947	0.904
Bands (LR)	Embodiment	–0.018	–0.049	0.961	12,010.492	12,020.980	–0.001
Delta (LME)	Embodiment	0.569	0.856	0.393	1,852.969	1,903.856	0.690
	Embodiment:Lobe_Central	–0.260	–0.339	0.735			
	Embodiment:Lobe_Parietal	–0.212	–0.277	0.782			
	Embodiment:Lobe_Temporal	–0.413	–0.539	0.591			
	Embodiment:Lobe_Occipital	–1.931	–2.518	0.012^*^			
Delta (LR)	Embodiment	0.168	0.390	0.697	2,044.538	2,051.808	–0.003
Theta (LME)	Embodiment	–0.462	–1.460	0.145	1312.026	1362.913	0.681
	Embodiment:Lobe_Central	0.282	0.990	0.323			
	Embodiment:Lobe_Parietal	0.667	2.341	0.020^*^			
	Embodiment:Lobe_Temporal	0.117	0.411	0.682			
	Embodiment:Lobe_Occipital	–0.210	–0.737	0.462			
Theta (LR)	Embodiment	0.112	0.713	0.476	1,479.939	1,487.209	–0.002
Alpha (LME)	Embodiment	0.256	0.487	0.627	1635.392	1,686.279	0.775
	Embodiment:Lobe_Central	–0.445	–0.902	0.368			
	Embodiment:Lobe_Parietal	–0.519	–1.053	0.293			
	Embodiment:Lobe_Temporal	–0.166	–0.337	0.736			
	Embodiment:Lobe_Occipital	0.468	0.948	0.344			
Alpha (LR)	Embodiment	–0.565	–1.786	0.075	1,872.141	1,879.411	0.008
Beta (LME)	Embodiment	–0.337	–1.186	0.237	1,296.811	1347.698	0.637
	Embodiment:Lobe_Central	0.224	0.785	0.433			
	Embodiment:Lobe_Parietal	0.152	0.535	0.593			
	Embodiment:Lobe_Temporal	0.222	0.780	0.436			
	Embodiment:Lobe_Occipital	0.818	2.871	0.004^*^			
Beta (LR)	Embodiment	–0.066	–0.443	0.658	1,448.874	1,456.144	–0.003
Gamma (LME)	Embodiment	–0.130	–0.437	0.663	1375.031	1425.918	0.617
	Embodiment:Lobe_Central	0.281	0.828	0.408			
	Embodiment:Lobe_Parietal	0.191	0.563	0.574			
	Embodiment:Lobe_Temporal	0.249	0.733	0.464			
	Embodiment:Lobe_Occipital	0.914	2.693	0.008^*^			
Gamma (LR)	Embodiment	0.261	1.512	0.132	1,532.812	1,540.082	0.005

The table includes the estimated effect of the embodiment score variable and its interaction with other variables in LME models, and the respective t-statistic, and *p*-value for each model. Variables that have a statistically significant effect (*p*-value < 0.05) on the outcome variable in the models are marked by an asterisk (“^*^”). Additionally, model performance metrics (AIC, BIC, and adjusted-R^2^) are provided to assess model fit.

**Figure 5:**
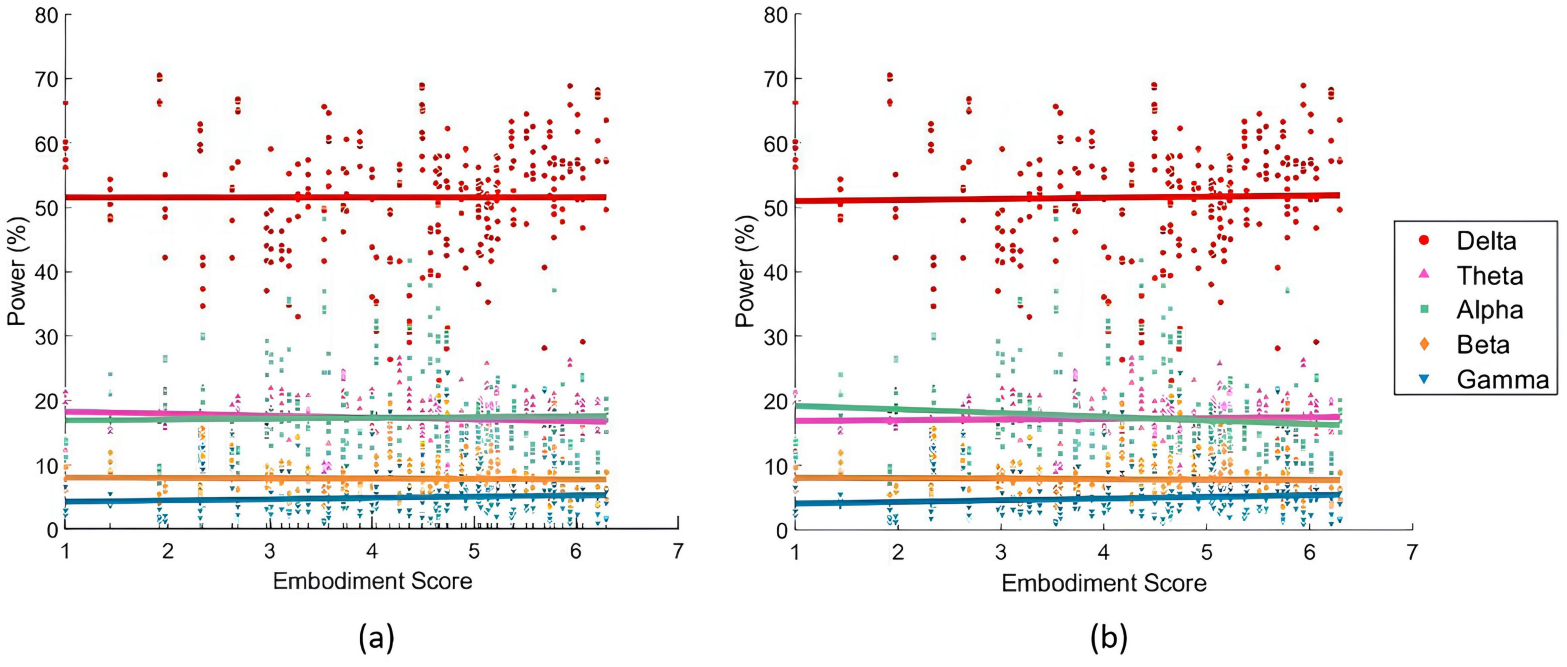
Relationships between embodiment score and EEG frequency power, divided in power bands. **(a)** Illustrates the partial dependence of embodiment score based on the results of the LME models (Equation 3 for the Delta band, Equation 4 for Theta, Alpha and Beta bands, and Equation 5 for Gamma band). **(b)** Presents the fitted LR models (each model based on Equation 1). The Delta band is represented in red, Theta in pink, Alpha in green, Beta in orange, and Gamma in blue.

When analyzing the SoE correlation with individual bands, the LME models consistently outperformed the LR models in fit (Table 4). Figure 5 compares both models' fits across different bands, showing the best LME model found for each band. Delta power was best described by Equation 3, which modeled it as a function of the interaction between embodiment score and lobes, with random intercepts for subjects. Theta, Alpha, and Beta bands were best captured by Equation 4, which extended the previous model by adding a random slope for condition at the subject level. Gamma power was most effectively modeled by Equation 5, which assumed an additive relationship among Gamma power, embodiment score, condition, and lobe, while accounting for random intercepts at the subject level. Regarding the correlations found, the LR models did not reveal significant interactions between frequency band power and embodiment scores. Similarly, the LME models did not identify direct correlations, but they did highlight significant interactions. Specifically, embodiment scores interacted significantly with the parietal lobe in predicting Theta power (*p*-value = 0.020; Table 4), and with the occipital cortex in predicting Delta (*p*-value = 0.012), Beta (*p*-value = 0.004), and Gamma (*p*-value = 0.008) powers (Table 4).


(3) $$\begin{array}{l} BandPower=EmbodimentScore*Lobe+\left(1|Subject\right) \end{array}$$

(4) $$\begin{array}{l} BandPower=EmbodimentScore*Lobe+\left(Condition|Subject\right) \end{array}$$

(5) $$\begin{array}{l} BandPower=EmbodimentScore+Condition+Lobe+\left(1|Subject\right) \end{array}$$

### 3.4 Machine learning models to predict embodiment

**Figure 6:**
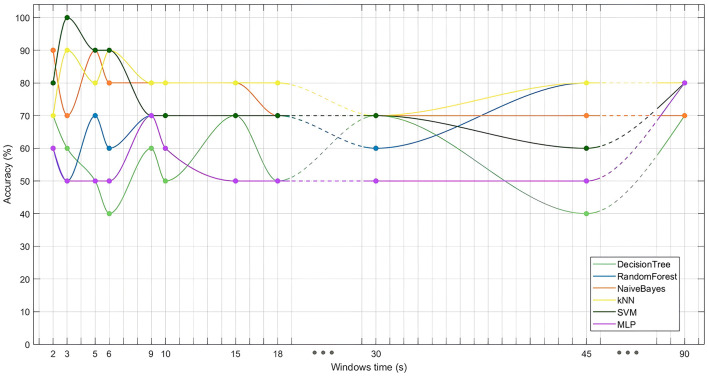
Performance (accuracy) of different classifiers (DT, RF, NB, kNN, SVM, and MLP) evaluated across various time window lengths tested (2, 3, 5, 6, 9, 10, 15, 18, 30, 45, and 90 seconds).

The performance of the binary classification models (Figure 6) revealed that the SVM model, using 3-second time windows, achieved an accuracy of 100%, highlighting the feasibility of distinguishing between embodiment states using machine learning algorithms. Interestingly, while the SVM performed best with smaller time windows, kNN models performed better with larger windows, though no model exceeded 80% accuracy with these larger windows. Overall, models using smaller time windows outperformed those with larger ones. Additionally, DTs, RFs and MLPs generally showed lower accuracy across most datasets.

**Figure 7:**
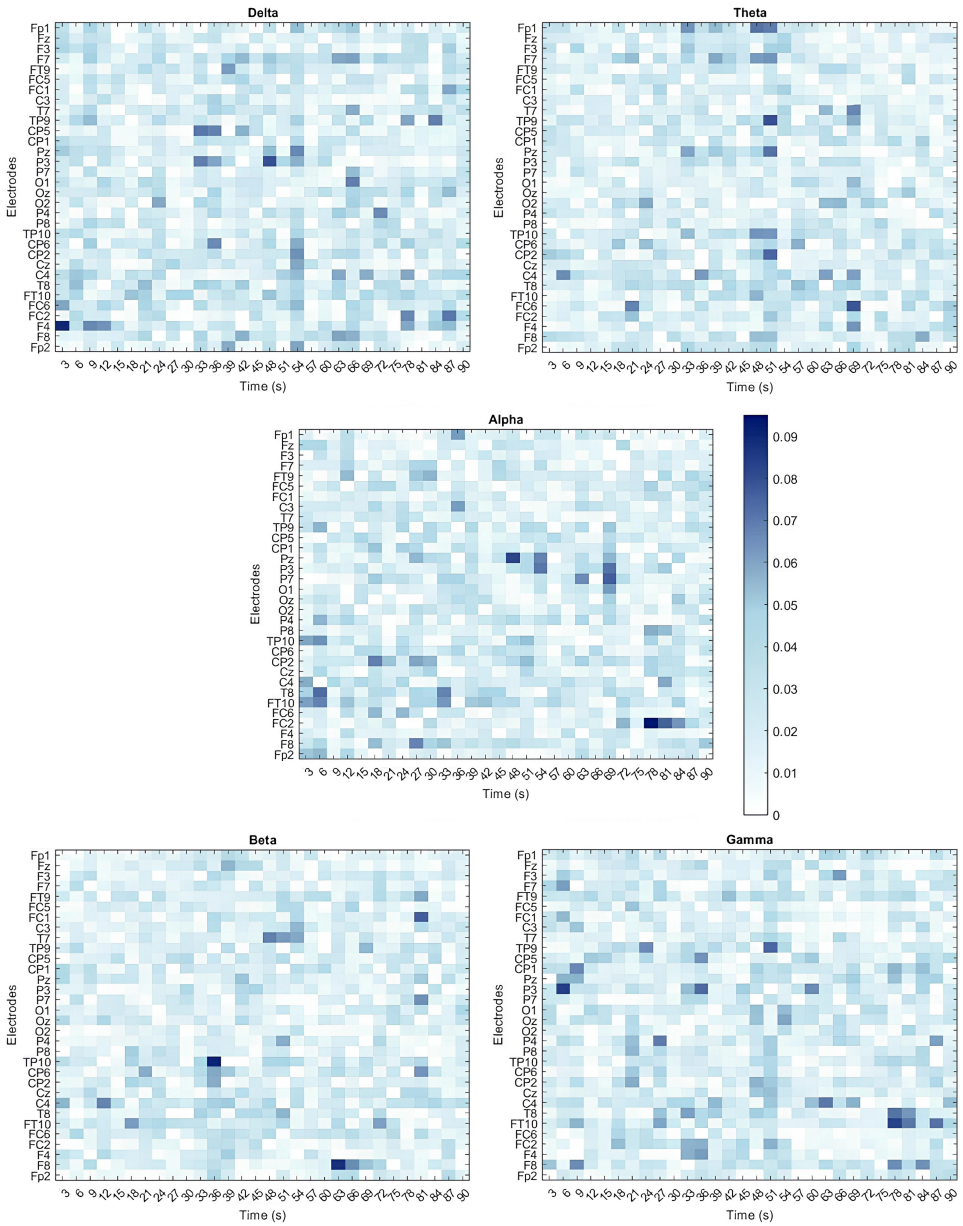
Heatmaps illustrating the importance of all 4,800 features for classifying embodiment using the SVM model trained on the 3-second time windows dataset. Each feature corresponds to the specific band power (Delta, Theta, Alpha, Beta, and Gamma) extracted from individual electrodes across the multiple time windows used. Feature importance is expressed as a percentage, with dark blue indicating the most important features and white representing the least important features.

Given the SVM model's strong performance in distinguishing between Embodied and Control conditions, particularly with 3-second time window signal segmentation, this model and dataset were chosen for feature importance analysis. Since the signal was segmented into 3-second windows, it resulted in 30 segments per electrode (90*seconds total signal* /3*seconds*). Therefore, the final SVM model used the 5 frequency bands extracted from all 30 segments across all 32 electrodes, leading to a total of 4,800 features (5 bands*30 segments*32 *electrodes*). The optimal hyperparameters for the model were identified as a linear kernel and a box constraint of 20.332. Feature importance was determined by the feature weights within the linear kernel, normalized to assess their contribution to classification. Since the input features were already standardized (using z-score normalization), the resulting weights were less biased by differences in scale. Heatmaps for the five frequency bands, shown in Figure 7, illustrate the spatial and temporal importance of features, while Table 5 lists the 15 most important features.

**Table 5:** The 15 most important features identified using the SVM model with 3-second time windows.

**Feature**	**Importance (%)**	**Feature**	**Importance (%)**	**Feature**	**Importance (%)**
FC2_Alpha_75-78s	0.093	FT10_Gamma_75-78s	0.083	FC2_Alpha_78-81s	0.077
TP10_Beta_33-36s	0.090	Pz_Alpha_45-48s	0.083	FC1_Beta_78-81s	0.076
F4_Delta_0-3s	0.089	P3_Delta_45-48s	0.080	P7_Alpha_66-69s	0.076
F8_Beta_60-63s	0.088	TP9_Theta_48-51s	0.079	CP2_Theta_48-51s	0.075
P3_Gamma_3-6s	0.084	FC6_Theta_66-69s	0.079	TP9_Gamma_48-51s	0.075

Each feature is labeled by its corresponding *Electrode, Band*, and *Time of the window* in which the spectral power contributing to the feature was extracted.

No clear spatial or temporal patterns emerged though some trends were observed. In the Delta band, features from earlier time windows showed greater importance in frontal areas (e.g., F4 Delta 0–3s), while later windows highlighted central (e.g., C4 Delta 30–36s; CP2 Delta 22–54s) and parietal regions (e.g., P3 Delta 45–48s). In contrast, Theta band features were more impactful in later time windows, particularly over temporoparietal and frontal areas (e.g., TP9 Theta 48–51s, FC6 Theta 66–69s). For the Alpha band, significant contributions were observed in fronto-central and parietal regions during the later stages of the illusion (e.g., FC2 Alpha 75–78s, Pz Alpha 45–48s, P7 Alpha 66–69s), and in the temporal lobe during initial stages (FT10, T8, TP10 electrodes). The Beta band also showed notable importance in later stages, particularly in temporoparietal and frontal regions (e.g., TP10 Beta 33–36s, F8 Beta 60–63s, FC1 Beta 78–81s). Finally, the Gamma band presented a more scattered importance over the scalp and time course. Still, it revealed early importance in the parietal lobe (e.g., P3 Gamma 3–6s) that shifted to temporal areas over time (e.g., FT10 Gamma 75–78s, P9 Gamma 48–51s). Despite these patterns, the features showed relatively low and similar importance, suggesting that focusing on a single band or time window is insufficient to capture the changes in brain activity associated with the SoE illusion.

## 4 Discussion

### 4.1 Effectiveness of the VR paradigm in inducing SoE

This study addressed the lack of standardized methodologies in embodiment research by following recent guidelines for inducing and disrupting the SoE through visuoproprioceptive, visuomotor, and visuotactile triggers. Prior research highlights the interplay of these sensory triggers in embodiment, showing that while visuoproprioceptive cues alone can induce SoE (Falcone et al., 2022; Carey et al., 2019), disrupting visuomotor or visuotactile feedback destabilizes it (Kokkinara and Slater, 2014). Synchronous feedback in one modality can compensate for asynchronous feedback in another (Huynh et al., 2019), suggesting that while a single trigger is sufficient, all contribute meaningfully in a non-hierarchical manner (Pritchard et al., 2016). The incorporation of all three triggers in this study ensured robust SoE induction. Additionally, SoE was assessed using a recent and validated questionnaire (Peck and Gonzalez-Franco, 2021) adding to its standardization.

The questionnaire results demonstrated strong SoE in the Embodied condition and its clear disruption in the Control condition (Figure 3), confirming the methodology's efficacy. Participants also reported a higher sense of presence in the Embodied condition, reinforcing shared sensory cues between presence and embodiment (Halbig and Latoschik, 2024; Pritchard et al., 2016). Furthermore, restricting hand movements for half the participants during the data acquisition did not significantly affect the illusion, since the results of the individual dataset (*p*-value = 0.001) and the combined one (*p*-value = 0.000) demonstrated the strong SoE induction in these participants. Thus, the results indicate that hand control is not the only relevant way to provide visuomotor cues, with head and torso synchronized movements alone providing sufficient visuomotor feedback. Therefore, this study stands out for its comprehensive approach, integrating validated questionnaires, all three sensory triggers, and a standardized 32-electrode EEG system. Many previous studies lack such methodological rigor, omitting key triggers or validated tools.

### 4.2 Changes in power spectrum during the SoE illusion

The frequency analysis revealed significant spatial and frequency differences between conditions, suggesting a potential biomarker for SoE (Table 3). Although the decrease in Delta power during SoE was not significant, it presents a novel finding, diverging from the typical focus on higher-frequency bands. Similarly, the overall reduction in Theta power suggests a potential link with virtual embodiment, aligning with prior research (Hansford et al., 2023; Jeunet et al., 2018). Regarding the Alpha band, its observed general reduction during the illusion, especially over the central and parietal cortex, is severely documented in the literature (Alchalabi et al., 2019; Raz et al., 2020; Evans and Blanke, 2013; Shibuya et al., 2021; Kang et al., 2015; Faivre et al., 2017; Rao and Kayser, 2017; Della Longa et al., 2021; Shibuya and Ohki, 2023; Sciortino and Kayser, 2022b,a; Shibuya et al., 2018). However, this study's lack of statistical significance raises doubts about Alpha's reliability as an SoE biomarker despite the apparent trend. Noteworthy, significant increases in the occipital Beta and the central and occipital Gamma power during the Embodied condition highlight these bands' role in SoE illusion.

Findings on Beta power align with Faivre et al. (2017), which reported its increased activity but in the frontal lobe, whereas this study identified occipital involvement. Methodological differences, including shorter SoE induction times (15 seconds vs. 110 seconds), may explain this discrepancy and suggest temporal dynamics in SoE as Beta activity may shift posteriorly over time. Some studies have reported Beta reductions during embodiment (Kang et al., 2015; Sciortino and Kayser, 2022b; Rao and Kayser, 2017), potentially due to non-VR setups or a focus on isolated SoE components rather than the full phenomenon. However, several studies observed Beta changes in overlapping regions, reinforcing its link to SoE. Regarding Gamma power, Hansford et al. (2023) found increases in the parietal lobe, while this study identified central and occipital involvement, likely due to different scalp division protocols. Similarly, Hiramoto et al. (2017) reported parietal Gamma increases during RHI with visuotactile stimulation, indicating that VR is not strictly necessary for such effects. Still, Li et al. (2023) also observed occipital Gamma increases in realistic VR scenarios, linking them to enhanced visual integration. Nonetheless, these findings reinforce the occipital lobe's role in SoE illusions and highlight the need for further spatial analysis. Additionally, the observed changes also align with theoretical models of SoE, where changes in brain power distributions reflect multisensory integration. The occipital lobe is crucial for integrating visual, proprioceptive, auditory, and tactile information (Beauchamp, 2005; Bertini et al., 2010), supporting visual perception, size integration, spatial transformations (Plewan et al., 2012; Weidner and Fink, 2007), visuo-haptic processing (Aman et al., 2010), and audiovisual object processing (Beer et al., 2013). Its significant activity changes suggest key multisensory contributions to embodiment. Beta activity is similarly linked to multisensory integration, coordinating vision and touch (Cooke, 2020), audiovisual stimuli (Ard et al., 2015), and sensory-motor synchronization (Senkowski et al., 2005). The observed occipital Beta increase likely reflects the brain's effort to synchronize sensory inputs for a cohesive virtual body experience. Gamma activity also plays a key role in multisensory alignment, particularly audiovisual synchronization (Senkowski et al., 2007). In the SoE context, Gamma oscillations help integrate visual, proprioceptive, and tactile cues, reinforcing their correct integration. Prior studies (Kanayama et al., 2009) have linked parietal Gamma activity to visuotactile integration during RHI, suggesting that central and occipital increases here serve a similar role in enhancing virtual body ownership.

Ultimately, these findings emphasize the role of immersive VR in engaging posterior brain regions during SoE. However, inter-subject variability, particularly from the Vagaja et al. (2024) dataset's between-subject design, poses challenges in identifying a definitive SoE biomarker. Such designs introduce significant variability, especially in frequencies below 40 Hz, due to anatomical and physiological differences (DelPozo-Banos et al., 2015; Kwak et al., 2023). Moreover, variability across studies underscores the impact of experimental design factors, such as VR immersiveness and scalp division protocols, on EEG correlates of SoE, reinforcing the need for standardized methodologies in the field.

### 4.3 Band power relationship with SoE strength

Linear models also revealed the importance of the occipital lobe in the SoE illusion. While total power analysis showed minimal direct association with embodiment (Table 4), individual bands revealed significant positive relationships between occipital Delta, occipital Beta, central Theta, and occipital Gamma, with SoE. This suggests the existence of critical band-specific relationships with SoE due to distinct frequency distributions (Figure 5). The lack of strong correlations with SoE directly highlights the complexity of SoE's neural mechanisms and emphasizes the importance of localized interactions and dynamic frequency changes rather than global metrics. Moreover, Beta and Gamma findings align with the frequency analysis, reinforcing their role in SoE. The significant central Theta correlation, previously undetected in condition comparisons, reinforces its observed trend of decreasing during SoE experiences, consistent with Pavone et al. (2016). These Delta and Theta relationships with SoE suggest subtle shifts in this band, which may not be strong enough to emerge in condition comparisons but still contribute to embodiment, underscoring the relevance of accounting for all bands in the SoE research rather than focusing only on higher frequency bands. These findings emphasize that SoE arises from dynamic interactions across multiple bands and regions rather than a single frequency predictor.

Nonetheless, models showed limited explanatory power, with high AIC/BIC values and low adjusted-R2 scores (Table 4), with LME models consistently outperforming LR models, likely due to their ability to account for random effects and inter-subject variability. This shows the importance of considering individual differences in SoE analyses. Additionally, it is important to note that each model in this study was treated as an independent analysis based on a priori hypotheses, and no multiple pairwise tests were performed. Therefore, no corrections for multiple comparisons (e.g., Bonferroni or FDR) across models were applied, although this could potentially increase the risk of Type I errors. This can represent a limitation in the statistical analysis and caution is warranted with the conclusions. Furthermore, it is important to acknowledge the limitation of correlating a subjective metric (questionnaire feature) with objective ones (band power). The questionnaire was used as a reference to assess embodiment; however, as previously discussed, it is not an ideal metric due to its susceptibility to personal factors, interpretation, and scale bias. Subjective and objective measures do not always align due to external influences, differences in scale types, and the distinct nature of these measurements. This misalignment could explain the lack of a linear relationship between SoE strength and frequency power, ultimately affecting the reliability of results. While no viable alternative for assessing SoE currently exists, the reliance on subjective measures remains a limitation, reinforcing the need for a standardized questionnaire approach to minimize biases and improve consistency in future studies.

### 4.4 Time-dependent changes in SoE illusions

Binary classification models further support the presence of a distinct brain pattern during SoE, as classifiers successfully differentiated between embodiment and non-embodiment conditions based on EEG frequency power (Table 3). While DT, RF, and MLP models performed poorly, particularly with smaller time windows, SVM models achieved the highest accuracy, reaching 100% with 3-second windows. The poor performance of DT, RF, and MLP likely stems from overfitting and difficulties handling high-dimensional data with limited samples. DT models also struggle with large feature sets and complex, non-linear relationships. These challenges highlight the impact of the feature-to-sample ratio, where a high ratio, as used in this study, significantly degrades model performance, especially in smaller datasets (Sammon et al., 1970; Hua et al., 2005). Thus, feature selection methods and larger sample sizes are relevant for improving model reliability.

Notably, smaller time windows consistently yielded higher classification accuracy (Figure 6), reinforcing the notion that SoE is a dynamic process over time. Aggregating data into larger windows may obscure temporal resolution, leading to performance reductions by averaging out transient neural activity. This temporal evolution of SoE has received limited exploration in the literature; still, it aligns with studies affirming that SoE has a rapid onset, emerges within seconds, and continues to strengthen over time (Kalckert and Ehrsson, 2017; Finotti et al., 2023; Perepelkina et al., 2018; Kondo and Sugimoto, 2022; Keenaghan et al., 2020). Moreover, the illusion's dependence on synchronized multisensory cues (Falcone et al., 2022) further contributes to its temporal variability, as disruptions in cue synchrony can destabilize perception (Kokkinara and Slater, 2014), potentially causing fluctuations in perception and corresponding brain activity.

Despite evidence of temporal evolution, feature importance analysis (Figure 7) in the best-performing model (SVM with 3-second windows) revealed no clear temporal or spatial dominance. Delta power was initially more relevant over frontal regions before spreading across the scalp, particularly in the centro-parietal cortex at later stages. Theta and Beta bands influenced later stages over fronto-central and temporoparietal areas, while Alpha power appeared in the parietal lobe early on before shifting to central regions. This further reinforce the association between the Alpha band over central and parietal areas and SoE, as literature affirms. Gamma power exhibited the most scattered pattern, with different features gaining importance at different times and brain regions. These findings suggest that SoE arises from distributed neural processes rather than a single dominant frequency or cortical region. No single lobe consistently dominates, pointing to a temporal evolution of lobe involvement, where specific lobes may play crucial roles at different stages of the illusion. The influence of the central lobe is supported by previous research emphasizing its involvement, particularly in the sensorimotor cortex (Kilteni et al., 2012; Frigeri et al., 2015). Several studies have documented changes in various bands over the central lobe during SoE illusions (Alchalabi et al., 2019; Raz et al., 2020; Evans and Blanke, 2013; Shibuya et al., 2021; Kang et al., 2015; Faivre et al., 2017; Rao and Kayser, 2017; Della Longa et al., 2021; Shibuya and Ohki, 2023; Sciortino and Kayser, 2022b,a; Shibuya et al., 2018; Pavone et al., 2016; Hansford et al., 2023; Li et al., 2023; Hiramoto et al., 2017). Furthermore, the frontal cortex's link to disembodiment and vestibular processing (Kilteni et al., 2012; Lopez et al., 2010), along with the temporal lobe's role in self-processing and multisensory integration (Arzy et al., 2006; Lenggenhager et al., 2006), reinforce their relevance to SoE. Structural connectivity studies also suggest that disruptions in frontal, parietal, and temporal connections may contribute to pathological embodiment, emphasizing the importance of these regions (Errante et al., 2022). Interestingly, while frequency analysis and linear models highlighted the occipital lobe's involvement in SoE, classification feature importance did not specifically emphasize it. This suggests that occipital activity may be more stable across time rather than restricted to specific time points, becoming more prominent in later stages of the illusion. These findings highlight the necessity of temporally sensitive analyses to fully capture the evolving neural dynamics of SoE.

Thus, SoE is a complex phenomenon involving widespread brain activity across frequency, temporal, and spatial domains. Despite its dynamic nature, its temporal evolution remains underexplored, underscoring the need for advanced analyses.

## 5 Conclusion

The findings confirm the effectiveness of the methods used to induce and assess SoE, establishing a standardized approach for SoE studies. Results reveal a distinct EEG frequency signature, with increased Beta and Gamma power in the occipital lobe strongly correlating with embodiment in later stages of the illusion. These bands, essential for multisensory integration and sensorimotor synchronization, emerge as promising SoE biomarkers. Machine learning models effectively classified SoE states, with SVM achieving the highest accuracy using 3-second windows, further validating robust neural markers. The study also highlights the temporal and spatial dynamics of SoE. Smaller time windows improved classification accuracy, suggesting SoE evolves over short timescales. No single frequency band or brain region exclusively defines SoE; instead, different EEG bands and lobes contribute at varying time points. Therefore, SoE emerges as a complex, dynamic process that evolves across temporal, frequency, and spatial domains. These findings offer valuable insights into its neural correlates and further reinforce its theoretical framework.

Despite these insights, several limitations should be acknowledged. The two-h data collection session may have affected participant focus, while the small sample size and mixed study design introduced variability. Future research should prioritize larger within-subject studies with shorter sessions to improve data quality and VE immersion. Refining machine learning models, such as incorporating feature selection to balance the feature-to-sample ratio and using the permutation-based feature importance method for a more scale-independent assessment of feature significance, could enhance performance, provide deeper insights into the contributions of individual features, and offer further understanding of SoE's temporal evolution. Then, this study focused on a lobe-level analysis; however, this approach may overlook some spatial specificity and potential lateralization effects. Therefore, future research should also consider electrode-level analysis to uncover finer-grained patterns. Additionally, advanced analyses, including time-frequency and connectivity-based approaches, are needed to identify more reliable EEG biomarkers. Standardized methodologies, higher electrode density, and multimodal imaging could further improve SoE biomarker identification.

## Data Availability

The datasets presented in this study can be found in online repositories. The names of the repository/repositories and accession number(s) can be found below: https://doi.org/10.5281/zenodo.8086086.

## Footnotes

^1^ https://github.com/noisys-project/Virtual-Embodiment-and-Motor-Imagery-BCIs

^2^ https://www.youtube.com/watch?v=txPpFjRKIos
